# Quality of life: a neglected aspect in male infertility studies

**DOI:** 10.1590/S1677-5538.IBJU.2026.9912

**Published:** 2026-06-30

**Authors:** Amrita Singh, Sandro C. Esteves

**Affiliations:** 1 University of Siena Department of Molecular and Developmental Medicine Siena Italy Master's Degree Programme in Biotechnology of Human Reproduction, Department of Molecular and Developmental Medicine, University of Siena, Siena, Italy; 2 Clínica de Andrologia e Reprodução Humana Campinas SP Brasil ANDROFERT, Clínica de Andrologia e Reprodução Humana, Campinas, SP, Brasil; 3 Universidade Estadual de Campinas Departamento de Cirurgia Campinas SP Brasil Departamento de Cirurgia (Divisão de Urologia), Universidade Estadual de Campinas (UNICAMP), Campinas, SP, Brasil; 4 Aarhus University Faculty of Health Aarhus Denmark Faculty of Health, Aarhus University, Aarhus C, Denmark

## INTRODUCTION

Male infertility is increasingly recognized not only as a reproductive disorder but also as a significant public health concern affecting the physical, emotional, social, and sexual well-being of affected individuals and their partners (1). While considerable attention has been devoted to understanding its causes, diagnostic pathways, and treatment options, relatively little emphasis has been placed on how infertility affects the daily lives of men and whether medical interventions meaningfully improve their quality of life (QoL) (2). This gap is particularly relevant given the growing recognition that patient-reported outcomes should complement traditional clinical endpoints such as semen quality, fertilization rates, pregnancy, and live birth.

The importance of this issue has been highlighted by the international Priority Setting Partnership for Infertility, which identified the psychological and emotional impact of infertility as one of the highest priorities for future research (3). Specifically, patients, healthcare professionals, and other stakeholders emphasized the need to understand how infertility affects emotional health and whether interventions addressing these concerns can improve patient outcomes. Despite these priorities, QoL remains one of the least investigated outcomes in male infertility research.

Although QoL has become an established outcome measure across multiple medical disciplines, there is no universally accepted definition. In the context of infertility, the concept of health-related quality of life (HRQoL) is particularly relevant because it encompasses the impact of disease and treatment on physical, psychological, emotional, social, and functional well-being (4). HRQoL recognizes that patients’ experiences extend beyond biological abnormalities and laboratory findings and are influenced by how disease affects their ability to live satisfying and productive lives.

## MEASURING QoL IN MALE INFERTILITY

Several generic and fertility-specific instruments have been developed to measure QoL in infertile individuals. Commonly used generic tools include the Short Form Health Survey (SF-36) and the World Health Organization Quality of Life Questionnaire (WHOQOL-BREF). Fertility-specific instruments include the Fertility Quality of Life questionnaire (FertiQoL), the Fertility Problem Inventory, and the Copenhagen Multi-Center Psychosocial Infertility (COMPI) Fertility Problem Stress Scales (1, 5-12) (Table-1). Among these, FertiQoL has become the most widely adopted instrument because it was specifically designed for infertile individuals and assesses emotional, social, relational, mind-body, treatment environment, and treatment tolerability domains.

**Table 1:** Tools to assess quality of life and infertility-related distress in individuals and couples affected by infertility.

Name	Characteristics	Website	Reference
**Generic**			
Short Form Health Survey (SF-36)	A 36-item questionnaire including physical functioning, role-physical, bodily pain, general health, vitality, social functioning, role-emotional and mental health.	Available from: https://www.clintools.com/victims/resources/assessment/health/sf36.html	9
World Health Organization Quality of Life (WHOQOL-BREF)	An abbreviated version of WHOQOL-100, including 26 items distributed into four domains (physical, psychological, social relationship, and environmental)	https://www.who.int/tools/whoqol	10
**Condition-specific**			
Copenhagen Multi-Center Psychosocial Infertility (COMPI) Fertility Problem Stress Scales	Fourteen items assessing the impact of infertility on personal, social, and marital domains. Items are scored using a four or five-point Likert scale, with higher scores indicating higher stress.	NA	8
Fertility Quality of Life (FertiQoL)	A 36-item questionnaire measuring fertility quality of life in four personal domains (emotional, social, relational, mind/body) and two treatment domains (tolerability, environment).	http://www.fertiqol.com/	11
Fertility Problem Inventory	A 46-item self-rating scale including five domains: social concern, sexual concern, relationship concern, need for parenthood, and rejection of a childless lifestyle. Participants respond on a 6-point Likert scale ranging from 1 to 6. A high item score indicates significant fertility-related stress.	NA	12

NA = not applicable

## PSYCHOLOGICAL BURDEN OF MALE INFERTILITY

Despite the availability of validated instruments, studies specifically addressing QoL in men with infertility remain scarce, although recent systematic reviews have emphasized that male infertility is consistently associated with impaired emotional, social, relational, and sexual well-being (13-15). Historically, infertility research has focused primarily on women because they bear most of the physical burden associated with assisted reproductive technology (ART). Consequently, the male experience has often been overlooked, despite evidence indicating that infertility can profoundly affect men's psychological health, sexuality, interpersonal relationships, social functioning, and self-perception (15).

Still, a few studies indicate that the QoL of men experiencing infertility appears to be dynamic and may evolve throughout the patient journey. Men may experience emotional distress during attempts to conceive, at the time of diagnosis, throughout infertility treatment, and particularly following unsuccessful ART cycles (16-18). The inability to achieve biological fatherhood may trigger a grieving process similar to that observed in other forms of loss. Furthermore, repeated treatment failures may compound emotional distress and contribute to treatment discontinuation (19). Studies have identified predictors of poorer QoL among infertile men, including lower educational attainment, unemployment, poor marital relationships, longer infertility duration, and previous failed IVF attempts (20-22). When the QoL of men with infertility is compared with infertile females, it has been observed that women consistently exhibit poorer FertiQoL scores than their male counterparts (16).

A 2024 systematic review evaluating the psychological consequences of male infertility highlighted the breadth of these effects (14). Men diagnosed with infertility frequently experience grief, loss, diminished self-esteem, feelings of inadequacy, anxiety, and depressive symptoms. The diagnosis may challenge deeply rooted perceptions of masculinity and fatherhood, leading some men to experience shame, social isolation, and stigmatization. Importantly, the review noted that men often internalize distress and are less likely than women to seek psychological support, potentially resulting in under-recognition of their emotional suffering. These observations reinforce the need for a patient-centered model of care that addresses the psychosocial consequences of infertility regardless of the underlying diagnosis.

## BEYOND SEMEN PARAMETERS: QoL, SEXUAL HEALTH AND HORMONAL FUNCTION

Interestingly, infertility-related QoL is not always closely linked to conventional reproductive biomarkers. One of the largest studies addressing this question evaluated more than 700 men participating in a multicenter randomized trial involving couples with unexplained infertility (23). The investigators examined relationships among fertility-related QoL, depression, gonadal function, semen quality, and erectile function. Contrary to expectations, neither QoL nor depressive symptoms were associated with testosterone concentrations or semen parameters. However, lower QoL scores were strongly associated with erectile dysfunction, and men with depressive symptoms were significantly more likely to experience erectile dysfunction than those without depression (Table-2). These findings suggest that psychological and sexual health dimensions may be more important determinants of perceived well-being than conventional biological markers of male reproductive function.

**Table 2:** Key primary studies evaluating quality of life in men with infertility.

Study	Population	Instrument	Main findings	Clinical implications
Coward et al., 2019 (23)	708 men from couples with unexplained infertility	FertiQoL, PHQ-9, IIEF	Lower QoL associated with erectile dysfunction but not testosterone levels or semen parameters	Psychological and sexual health may be more relevant determinants of QoL than biological markers
Asazawa et al., 2019 (33)	38 infertile couples	FertiQoL	Spousal-support program improved emotional and relational QoL domains	Psychosocial interventions may improve patient well-being
Danis et al., 2023 (27)	201 men undergoing fertility evaluation	FertiQoL	Men evaluated by reproductive urologists reported lower QoL but overwhelmingly considered the consultation beneficial	Supports specialist male infertility evaluation and patient education
Pan et al., 2026 (26)	105 couples undergoing ART	FertiQoL	Female infertility negatively affected male partner QoL; infertility operates as a dyadic stressor	Supports couple-centered infertility care

The observations discussed above carry important clinical implications. Physicians often focus on semen quality and endocrine parameters when evaluating infertile men, yet these measures may inadequately capture the patient's lived experience. Men with relatively preserved semen parameters may still suffer substantial psychological burden, whereas others with severe abnormalities may report acceptable QoL. Consequently, QoL assessment may provide clinically relevant information that is not apparent from traditional diagnostic testing (24, 25).

The relationship between infertility and sexual health deserves particular attention. Erectile dysfunction reduced sexual satisfaction, and diminished sexual confidence have been reported more frequently among infertile men than fertile controls (23). While these disturbances may partly reflect underlying hormonal or vascular conditions, psychological distress appears to play a major role. The association between lower QoL and erectile dysfunction observed in infertile men suggests that sexual and emotional health should be routinely assessed during infertility evaluations. Recognition of these issues may create opportunities for interventions capable of improving overall well-being independently of reproductive outcomes.

## INFERTILITY AS A DYADIC DISEASE

Another emerging concept is that infertility should be regarded as a dyadic condition rather than an individual disease. Although infertility diagnoses are traditionally assigned to one partner, the consequences are often experienced by both members of the couple. Recent studies using actor-partner interdependence models have provided important insights into these interactions (26). In a study involving couples undergoing ART, female infertility was associated with significantly lower QoL among male partners, particularly in emotional and mind-body domains (Table-2). Interestingly, the converse effect was not observed. These findings suggest that men may experience substantial distress when witnessing the physical and emotional burden of treatment imposed on their partners, even when the infertility diagnosis is not attributed to them. Such observations challenge traditional assumptions regarding gender differences in infertility experiences and emphasize the need for couple-centered care models.

## THE ROLE OF SPECIALIST MALE INFERTILITY CARE

Additional evidence supporting the importance of male-centered infertility care comes from studies examining fertility-related QoL according to provider type. Men evaluated by reproductive urologists have been reported to exhibit lower FertiQoL scores than those evaluated exclusively by reproductive endocrinologists (27) as it likely reflects referral pathways and case complexity rather than differences in quality of care. Men referred for reproductive urology assessment often have already undergone reproductive endocrinology evaluation and may present with greater diagnostic uncertainty, disease burden, or treatment-related distress.

Importantly, the same study suggested that men seen by reproductive urologists felt more informed and better understood regarding their condition, underscoring the potential value of male-focused evaluation despite the higher distress observed in this subgroup (27) (Table-2). These findings suggest that specialist male infertility evaluation may provide benefits extending beyond diagnosis and treatment, including education, reassurance, empowerment, and improved health awareness (2, 28).

The latter point is particularly relevant because male infertility is increasingly recognized as a marker of overall health. A growing body of evidence indicates that infertile men face higher risks of metabolic disorders, hypogonadism, cardiovascular disease, malignancies, and reduced life expectancy (29). Consequently, infertility evaluation represents a unique opportunity to identify previously undiagnosed health conditions and promote preventive healthcare strategies. From a QoL perspective, improved understanding of one's health status and access to appropriate treatment may yield benefits extending far beyond reproductive outcomes.

This broader view of male infertility has been reinforced by the recently published World Health Organization (WHO) Infertility Guidelines, which emphasize that infertility care should be delivered as an integrated component of sexual and reproductive healthcare across the life course (30-32). Importantly, the WHO recognizes that infertility affects both members of the couple and recommends that men and women undergo concurrent evaluation rather than the traditional sequential approach in which the female partner is often investigated first and the male partner only later, particularly when assisted reproductive technologies are being considered. The guideline highlights that infertility assessment should begin with a comprehensive medical, reproductive, and sexual history, focused physical examination, and semen analysis performed according to WHO standards. Such an approach acknowledges the substantial contribution of male factors to infertility and seeks to avoid delays in diagnosis and treatment.

## CAN QoL BE IMPROVED?

Despite increasing recognition of the psychosocial burden of infertility, remarkably little research has evaluated interventions designed to improve QoL among infertile men. For example, the effects of varicocele repair, hormonal therapies, sperm retrieval procedures, sperm DNA fragmentation management strategies, lifestyle interventions, and ART itself on patient-reported QoL outcomes remain poorly characterized. Consequently, clinicians often lack evidence regarding whether treatments that improve reproductive outcomes also improve patients’ lives.

One notable exception is a pilot study investigating a structured spousal-support program for men undergoing infertility treatment (Asazawa et al. 2020) (33) (Table-2). The intervention significantly improved emotional and relational QoL domains and was well accepted by participants. Although preliminary, these findings suggest that psychosocial and educational interventions may represent promising strategies for alleviating infertility-related distress. In another study, treating male hypogonadotropic hypogonadism with gonadotropins improved scores of all SF-36 QoL domains (34).

The paucity of intervention studies represents one of the most important gaps in current knowledge. This gap is particularly concerning because patients frequently value outcomes extending beyond conception and live birth. Many seek explanations for their infertility, reassurance regarding their health, and opportunities to regain a sense of control over their reproductive future. In some cases, successful diagnosis and treatment may improve QoL even when pregnancy is not achieved. Conversely, interventions associated with significant physical, emotional, or financial burdens may adversely affect QoL despite improving reproductive outcomes. These considerations underscore the importance of incorporating patient-reported outcome measures into both clinical practice and research (24, 25).

Notwithstanding the limited data about the impact of male infertility treatment on QoL, it has been recognized that education provision and proper andrological evaluation provide a unique opportunity for identifying correctable conditions causing infertility and health-threatening issues or coexistent diseases that require medical attention (such as obesity, metabolic syndrome, erectile dysfunction, hypogonadism, kidney diseases, and cancer) (2, 35, 36). Still, in real-world settings, most men are poorly educated about infertility risk factors and do not follow basic recommendations for a healthy lifestyle (37). This is aggravated by the fact that the clinical evaluation of men having infertility is suboptimal, particularly in IVF clinics, where about 40% of specialists report taking only a brief medical history and 24% reported that they never perform a physical examination (38).

Future studies should therefore move beyond traditional reproductive endpoints and routinely incorporate validated QoL instruments. Such efforts would provide a more comprehensive understanding of the impact of infertility and its treatment on affected individuals and couples. Longitudinal studies are particularly needed to characterize changes in QoL over time and to identify factors predicting resilience or vulnerability. Randomized trials evaluating medical, surgical, lifestyle, educational, and psychosocial interventions should determine not only whether these interventions improve fertility outcomes but also whether they enhance patients’ overall well-being.

## CONCLUSIONS

Quality of life remains a neglected outcome in male infertility research despite growing recognition of its clinical importance. Available evidence indicates that infertility can profoundly affect psychological health, sexual function, interpersonal relationships, and overall well-being. Emerging data further suggest that infertility should be viewed as a shared condition affecting both partners and that specialist male infertility evaluation may provide meaningful benefits beyond reproductive diagnosis alone. Nevertheless, substantial knowledge gaps remain regarding the mechanisms linking infertility and QoL and, more importantly, regarding interventions capable of improving patient well-being. Addressing these gaps will be essential for advancing truly patient-centered infertility care and ensuring that treatment success is measured not only by reproductive outcomes but also by improvements in the lives of the men and couples we serve.

## Data Availability

All data generated or analysed during this study are included in the published article.
